# PPG2EMG: Estimating Upper-Arm Muscle Activities and EMG from Wrist PPG Values

**DOI:** 10.3390/s23041782

**Published:** 2023-02-05

**Authors:** Masahiro Okamoto, Kazuya Murao

**Affiliations:** Graduate School of Information Science and Engineering, Ritsumeikan University, 1-1-1 Nojihigashi, Kusatsu, Shiga 525-8577, Japan

**Keywords:** wearable computing, sensing, pulse wave, electromyogram, PPG, EMG, muscle activity, activity recognition

## Abstract

The electromyogram (EMG) is a waveform representation of the action potential generated by muscle cells using electrodes. EMG acquired using surface electrodes is called surface EMG (sEMG), and it is the acquisition of muscle action potentials transmitted by volume conduction from the skin. Surface electrodes require disposable conductive gel or adhesive tape to be attached to the skin, which is costly to run, and the tape is hard on the skin when it is removed. Muscle activity can be evaluated by acquiring muscle potentials and analyzing quantitative, temporal, and frequency factors. It is also possible to evaluate muscle fatigue because the frequency of the EMG becomes lower as the muscle becomes fatigued. Research on human activity recognition from EMG signals has been actively conducted and applied to systems that support arm and hand functions. This paper proposes a method for recognizing the muscle activity state of the arm using pulse wave data (PPG: Photoplethysmography) and a method for estimating EMG using pulse wave data. This paper assumes that the PPG sensor is worn on the user’s wrist to measure the heart rate. The user also attaches an elastic band to the upper arm, and when the user exerts a force on the arm, the muscles of the upper arm contract. The arteries are then constricted, and the pulse wave measured at the wrist becomes weak. From the change in the pulse wave, the muscle activity of the arm can be recognized and the number of action potentials of the muscle can be estimated. From the evaluation experiment with five subjects, three types of muscle activity were recognized with 80+%, and EMG was estimated with approximately 20% error rate.

## 1. Introduction

The electromyogram (EMG) [1] is a waveform representation of the action potential generated by muscle cells using electrodes. Action potentials are mainly acquired with needle, wire, and surface electrodes, and the type and role of the EMG differ depending on the electrode used. In particular, EMG acquired using surface electrodes is called surface electromyography (sEMG), and it is the acquisition of muscle action potentials transmitted by volume conduction from the skin. Surface electrodes are approximately 5–10 mm in diameter and are used by attaching them directly to the skin; they can measure a wide range of action potentials close to the body surface between two electrodes. Needle electrodes are mainly divided into concentric needle electrodes and single muscle fiber needle electrodes. The concentric needle electrode can measure action potentials generated by muscle fibers within approximately 0.5 mm of the needle tip, while the single muscle fiber needle electrode has a smaller measurement range than the concentric needle electrode and can measure action potentials generated by a single muscle fiber. Needle electrodes are mainly used to diagnose neuromuscular diseases and can be painful when measuring muscle activity. A wire electrode is a soft, hair-thin electrode that can be inserted into the muscle with a needle to measure action potentials at a greater depth than a surface electrode. Various types of wire electrodes depend on the metal used as the wire, such as platinum and stainless steel wire, and they differ in characteristics such as ease of wire disconnection and wire extraction. The wire electrode is a thin and flexible material, which has the advantage of not causing pain when measuring muscle activity, unlike the needle electrode.

Compared to needle and wire electrodes, surface electrodes can be used by simply attaching the electrodes to the skin, and have the advantage of being painless. However, surface electrodes require disposable conductive gel or adhesive tape to be attached to the skin, which is costly to run, and the tape is hard on the skin when it is removed. In addition, the burden on the user is greater because multiple electrodes need to be attached to recognize a person’s activity and state. On the other hand, Matsuhisa et al. [2] have fabricated a sensor that can measure muscle potential by printing a circuit on fabric using conductive ink and wearing it, which can be attached to clothing to measure muscle potential over a long period. However, it is cumbersome to wear the EMG sensor, which is not worn daily, to measure the EMG potential, and requires additional power consumption, wiring, communication volume, and storage.

Muscle activity can be evaluated by acquiring muscle potentials and analyzing quantitative, temporal, and frequency factors. For example, the amount of muscle activity can be evaluated by analyzing the amplitude of the EMG potential. Joint abnormalities can be detected from the gap in time when the EMG potential becomes active in each muscle during exercise. It is also possible to evaluate muscle fatigue because the frequency of the EMG becomes lower as the muscle becomes fatigued. Research on human activity recognition from EMG signals has been actively conducted, and has been applied to systems that support arm and hand functions, such as power assist devices [3]. In addition, as a new input method to replace the conventional keyboard and mouse input, a method to recognize finger and arm gestures based on EMGs has been proposed [4,5,6]. As described above, myoelectricity has been applied in various fields.

This paper proposes a method for recognizing the muscle activity state of the arm using pulse wave data and a method for estimating EMG using pulse wave data. This paper assumes that the pulse wave (PPG: Photoplethysmography) sensor is worn on the user’s wrist to measure the heart rate. The user also attaches an elastic band to the upper arm, and when the user exerts a force on the arm, the muscles of the upper arm contract. The arteries are then constricted, and the pulse wave measured at the wrist becomes weak. The strength of the pulse wave is expressed as an increase or decrease in amplitude. From the change in the pulse wave, the muscle activity of the arm can be recognized, and the number of action potentials of the muscle can be estimated. The method for recognizing the arm muscle activity state using pulse wave data discriminates between four states of armload from pulse wave sensor measurements. In the method of estimating the amount of muscle activity using pulse wave data, the root mean square of the muscle potential is regressed from the pulse wave sensor measurements. In this paper, surface electrodes are used, which do not require a needle and can acquire EMG simply by attaching the electrode to the skin, because we do not need to measure local muscle fibers as in the case of needle and wire electrodes.

The proposed method has the following three features.

- Easy to wear: Compared to an EMG sensor, there is no need for multiple electrodes to be attached, and no gel or adhesive tape is used, resulting in less stress on the skin.
- Low cost: All you need is a rubber band (≤1 USD). Disposable conductive gel and adhesive tape are not used.
- No additional equipment: Only a smartwatch or activity meter equipped with a PPG sensor is needed. There is no need for wiring or communication ports for the muscle action potential sensing. Data size and power consumption do not increase.

This paper is organized as follows. Section 2 introduces related work. The proposed method is explained in Section 3 and the evaluation experiments are described, and the results are discussed in Section 4. The challenges of the proposed method and its solutions are discussed in Section 5, and finally, Section 6 summarizes the paper.

## 2. Related Work

This section introduces studies on human activity recognition using EMG or PPG.

### 2.1. Human Activity Recognition Using EMG

Zhang et al. [7] used an accelerometer on the user’s wrist and an EMG sensor on the forearm to recognize sign language words and sentences, as well as 18 different Rubik’s Cube operations. The algorithm is based on a combination of decision trees and multi-stream hidden Markov models, and automatically extracts the start and end points of the gesture from the EMG values. Kurosawa et al. [8] presented the MyoTilt target selection method for smartwatches, which employs a combination of a tilt operation and EMG. EMG senses the force and moves the cursor in the direction where the user tilts the arm to manipulate the cursor. In this way, the user can manipulate the cursor on the smartwatch with minimal effort, by tiling the arm and applying force to it. Participants selected small targets with an accuracy greater than 93.89%. Huang et al. [4] proposed a system that recognizes thumb gestures from forearm myoelectricity. Since a large amount of training data is required to classify minute thumb movements, the training data is obtained from thumb movements that users perform in their daily lives. McIntosh et al. [5] used myoelectric and pressure sensors attached to the forearm to recognize 15 different gestures using the hand and fingers. They showed that myoelectricity is suitable for detecting finger gestures, that pressure is suitable for detecting wrist and forearm rotation, and that combining both sensors increases the recognition accuracy. Saponas et al. [9] proposed a method for recognizing finger pinch gestures such as pinching in the air and pushing in space based on myoelectricity in the upper part of the forearm and showed a recognition accuracy of 86% for pinching gestures and 76% for pushing in space. They have also developed a system that does not require new training or calibration after the sensor is reattached. Amma et al. [6] used an electrode array with 192 electrodes mounted on the upper forearm to perform 27 different finger gesture recognition, showing a recognition accuracy of 90%. In an environment where the training and test data were collected on different days, the recognition accuracy was 59% due to a slight change in the sensor mounting position. Applying an algorithm to correct the sensor mounting position improved the recognition accuracy to 75%. Duente et al. [10] proposed a system in which a muscle potential sensor and an electric muscle stimulation (EMS) device are attached to the forearm, notifications from a smartwatch are transmitted via EMS, and the user can choose to receive or reject notifications based on muscle potential values. The response error rate of this system is 3.9%, which is faster than the hand-operated interaction with the smartwatch. Javaid et al. [11] proposed the classification and recognition of hand gestures using EMG signals for controlling the upper limb prosthesis. The EMG signals were measured through a band of MYO gesture control. The EMG data were acquired from 10 healthy subjects (five males and five females) performing four upper limb movements. The execution of these classifiers shows an overall accuracy of 83.9%.

### 2.2. Human Activity Recognition Using PPG

Havriushenko et al. [12] proposed using neural networks to estimate a user’s respiratory rate from pulse wave data. The respiratory rate is often measured with a thermal sensor placed in the nasal channels or an elastic chest belt, but these devices may interfere with sleep. In contrast, their method can be implemented in a wearable device. Their evaluation showed an average respiratory rate estimation error lower than 2.2 breaths per min. Jarchi et al. [13] proposed a method that relies on a nonlinear time-frequency representation, called the wavelet synchrosqueezed transform (WSST), to estimate the instantaneous respiratory rate from body-mounted PPG sensors. Han et al. [14] proposed a method for detecting premature atrial contraction and ventricular contraction using PPG data from a smartwatch. Wang et al. [15] developed a system for identifying excess alcohol consumption using an SVM with ECG and PPG monitoring data. Longmore et al. [16] sought to identify a single location in the human anatomy for measuring the heart rate (HR), blood oxygen saturation (SpO2), and respiration rate at rest simultaneously and while walking by a single PPG sensor.

Among potential pulse data applications related to emotions, Goshvarpour et al. [17] proposed a method for classifying emotional responses using a simple dynamic signal processing technique and a fusion framework. They recorded the ECG and finger pulse activity of 35 subjects during a rest condition and when the subjects were listening to music intended to stimulate certain emotions. After constructing Poincaré plots, an SVM was used to classify them into four emotions: happiness, sadness, peacefulness, and fear. Kajiwara et al. [18] developed an application for logistics companies that adopt a manual order-picking system, given that emotions and engagement affect work efficiency and human errors. Specifically, they proposed a method for predicting emotions and engagement during work with a high exercise intensity from behavior and pulse wave data acquired by wearable devices. Pulse waves, eye movement, and general movement data are input to deep neural networks to estimate a worker’s emotion and engagement. The results of verification experiments showed that the emotion and engagement during order picking could be accurately predicted from the worker’s behavior with an error rate of 0.12 or less. Lee et al. [19] researched improving the speed of emotion recognition by using a PPG signal. A two-dimensional emotion model based on valence and arousal was adopted, and a one-dimensional convolutional neural network (1D CNN) was used to recognize emotions from a 1.1-s PPG signal. The 1D CNN was tested as a binary classifier (high or low valence and arousal) using the dataset for emotion analysis using physiological signals (DEAP). It achieved recognition accuracies of 75.3% for valence and 76.2% for arousal. Udovičić et al. [20] studied emotion recognition using only GSR and PPG signals because of their suitability for implementation in a simple wearable device that can collect signals from a person without compromising comfort and privacy. In addition to the above studies, there have been many others on the use of PPG data for emotion recognition [21,22,23].

Kotzen et al. [24] developed SleepPPG-Net, a DL model for 4-class sleep staging from the raw PPG time series. SleepPPG-Net was trained end-to-end and consists of a residual convolutional network for automatic feature extraction and a temporal convolutional network to capture long-range contextual information. When benchmarked on a held-out test set, SleepPPG-Net obtained a median Cohen’s Kappa score of 0.75 against 0.69 for the best SOTA approach. Yoshida et al. [25] proposed a method for estimating the load position of wearable devices using pulse waves and ECG. They estimate the arrival time of the pulse wave at the wearable device mounting position by comparing the heartbeat obtained by the ECG sensor with the pulse wave obtained by the pulse wave sensor at load position and estimate the load position of wearable devices by calculating the KL divergence between the distribution of the estimated time and the distribution of the training data collected at each site in advance. Akimoto et al. [26] proposed an interaction method for wearable devices that executes simple commands by sensing changes in blood flow caused by pressure on the body. Pulse waves are measured with a smartwatch on the wrist, and the pulse wave peaks are detected from the acquired data. The time difference between the peaks is used to obtain the time when the upper arm is compressed, and commands are executed according to the compression time. Methods for recognizing human states have been proposed because the heart rate changes due to sleep and tension. In addition, it is possible to obtain the time spent exercising simply by measuring the heart rate. However, as far as we know, no research focuses on the fact that the pulse wave changes due to muscle activity and estimates the amount of muscle activity.

## 3. Proposed Method

This section explains the proposed method that recognizes muscle activity states and estimates EMG from pulse wave data.

### 3.1. Principle

Muscle activity can be obtained by analyzing muscle potential, and the amount of muscle activity appears as the amplitude of the muscle potential. When muscle activity becomes active, the amplitude of the EMG becomes large, and when muscle activity decreases, the amplitude of the EMG becomes small. If external pressure is applied to the artery, the pulse wave passing through the artery will be attenuated [27,28]. If an elastic band is attached to the upper arm of the arm wearing a smartwatch, blood vessels in the upper arm are compressed by the band as the muscles in the upper arm contract such as by bending the arm. The pressure applied to the blood vessels in the upper arm is small when the arm is stretched, and the muscles are not contracted, while greater pressure is applied to the blood vessels when a greater load is given to the muscles, such as lifting heavy objects. Therefore, by measuring pulse waves with a PPG sensor mounted on a smartwatch or activity meter worn on the wrist, the state of the upper arm and the amount of muscle activity can be estimated. Even when the elastic band is not worn on the upper arm, the pulse wave is attenuated only by arterial compression by muscles, however, since the effect is small and individual and environmental differences are large, this study assumes that the band is worn. Focusing on the fact that differences in muscle activity appear in the pulse wave, this paper proposes a method for recognizing multiple pre-defined states of arm muscle activity from pulse wave data (classification problem) and a method for estimating arm EMG from pulse wave data (regression problem).

### 3.2. Muscle Activity Recognition Method

The proposed system collects PPG data p(t) at time *t* through a PPG sensor attached to the user’s wrist. Then fast Fourier transform (FFT) is applied to p(t) over *N*-sample length window and X(k) is obtained. The sampling frequency of PPG is set to 100 Hz and *N* is set to 200 (2 s).

(1) $$X\left(k\right)=\sum_{t=T-N+1}^{T}p\left(t\right)\exp\left(-j\frac{2\pi kt}{N}\right)$$

Then, power spectrum P(k) is obtained from X(k) by $P\left(k\right)={\left|X\left(k\right)\right|}^{2}$.

(2) $$P\left(k\right)={\left|X\left(k\right)\right|}^{2}$$

In the training phase, P(k) are collected for each pre-defined class of muscle activities and annotated with the class label, then the classifier is trained with the labeled data. In the testing phase, P(k) of the unknown muscle activity is fed to the model, and the classification result is obtained. In this paper, a random forest is used as the classifier.

### 3.3. EMG Estimation Method

**Figure 1:**
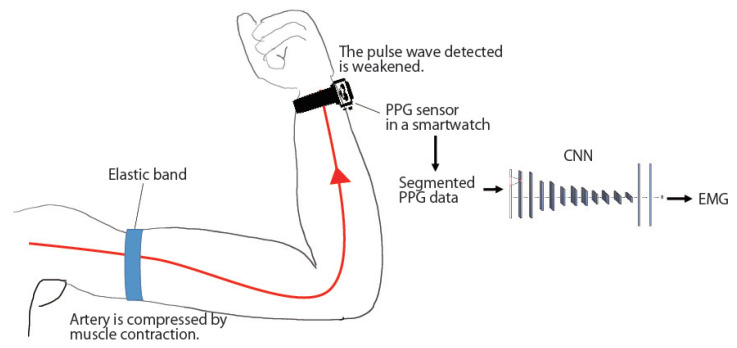
Flow of the EMG estimation method.

The flow of the EMG estimation is shown in Figure 1. The proposed system collects PPG data p(t) through the PPG sensor and EMG data s(t) through electrodes attached to the upper arm at time *t*. Note that training data consists of PPG and EMG data, while the testing data is only PPG data. The PPG and EMG data sampling frequency is set to 100 Hz and 1000 Hz, respectively.

At first, root mean square (RMS) r(T) is calculated at time t=T from the EMG data s(t) over 10N-sample length window. RMS is the square root of the mean value of the squared amplitude. RMS of EMG is commonly used to evaluate muscle activity [29,30,31]. In this paper, *N* is set to 200 (2 s).

(3) $$r\left(T\right)=\sqrt{\frac{1}{10N}\sum_{t=T-10N}^{T}s{\left(t\right)}^{2}}$$

**Figure 2:**
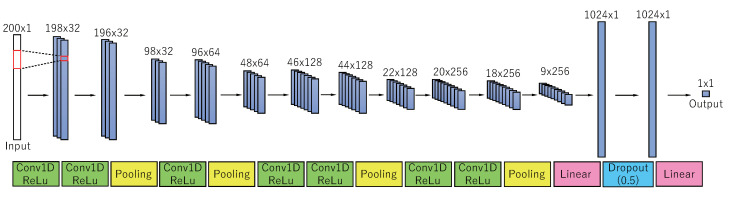
CNN for regression of RMS of EMG.

Then, pulse wave [p(T−N+1),⋯,p(T)] over *N*-sample window at time t=T annotated with r(T) is fed into the convolutional neural network (CNN) for training. Figure 2 shows our CNN. In our CNN, kernel_size of convolution layer is 3, the activation function is Rectified Linear Unit (ReLU), pool_size of Pooling layer is 2, the number of the hidden layer is 128, and the dropout rate is 0.5. L1 loss is used as a loss function. An optimization method is Adam. Batch size *B* is set to 64.

(4) $$L\left(y(T),r(T)\right)=\frac{1}{B}\sum_{i=1}^{B}\left|y_{i}\left(T\right)-r_{i}\left(T\right)\right|,$$

where r(T) is true RMS of EMG (groundtruth) over 10N samples, y(T)∈R is outout of CNN, *B* is batch size, $y_{i}\left(T\right)$ is r(T) in *i*-th batch, and $r_{i}\left(T\right)$ is r(T) in *i*-th batch.

## 4. Evaluation

A preliminary experiment was conducted to investigate the effect of the compression of the upper arm on the pulse wave. Then, the performance of the proposed methods for muscle activity recognition and EMG estimation was evaluated.

### 4.1. Preliminary Experiment

This section describes the preliminary experiment conducted to investigate the effect of the compression of the upper arm on the pulse wave caused.

#### 4.1.1. Setup

One of the authors (male, 23 years old) joined the experiment. The subject attached a PPG sensor to the right wrist while compressing the upper arm with a tourniquet (rubber band). When the arm is not bent, the tourniquet is not tight enough to prevent it from slipping off the arm. The PPG data was collected on a PC through Arduino Uno. A pressure sensor is also attached between the tourniquet and the skin. The pressure value applied to the upper arm by the compression is recorded just for reference and is not used in the evaluation.

**Figure 3:**
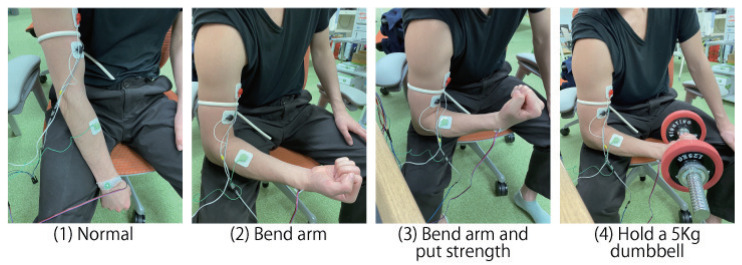
Four types of state using muscle.

Pulse wave data were collected in four states shown in Figure 3; (1) Normal: the subject is putting down the arm, (2) Bend arm: the subject is bending the arm, (3) Bend arm and put strength: the subject is bending the arm and putting strength into the upper arm, and (4) Hold a 5 Kg dumbbell: the subject is bending the arm with holding a 5 Kg dumbbell. Each state was maintained for 10 s. After a 10-s measurement, the arm was returned to state (1) and maintained again for 10 s. The procedure was repeated 60 times for 10 s in each state, and a total of 40 min of data (=10 s × 60 times × 4 states × 1 persons) was collected. Sixty data sets were split into training and test data in a 10-fold cross-validation manner. For each training and test data, the power spectrum for the collected data was calculated over a 2-s window with a 50% overlap sliding window. One 10-s data produces 9 sets of power spectrum data. A total of 2160 samples (= 9 sets × 60 times *×* 4 states) were prepared.

#### 4.1.2. Result

**Figure 4:**
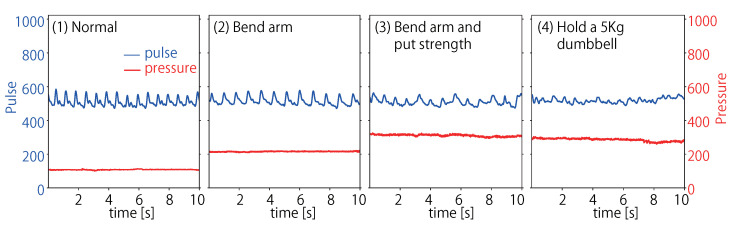
PPG data and pressure data collected in four states.

At first, the relationship between wrist PPG and upper arm pressure is investigated. Figure 4 shows PPG data and the pressure data received by the upper arm from the tourniquet. The pressure on the upper arm due to the tourniquet is the smallest in (1), the next smallest in (2), and the largest in (3) and (4). These values are consistent with the intuitive magnitude of muscle activity. Regarding the PPG, the peak shape of the pulse wave is clearly expressed in (1) and (2), but the peak of the waveform in (3) is low, and it is difficult to distinguish the peak when holding a dumbbell in (4). In addition, the pulse wave in (1) descends rapidly after reaching its peak, while in (2) the pulse wave descends gently after reaching its peak. When pulse waves were additionally measured with the arm stretched out horizontally, it was confirmed that they descended gently after reaching the peak as in (2). From this, it can be considered that the pulse wave in (1) was affected by gravity due to the downward extension of the arm. In other words, it may be possible to classify whether the arm is stretched horizontally or downward even in the normal state. In state (3), it was difficult to exert the same force each time during the experiment, and the amplitude and shape of the pulse wave varied.

**Figure 5:**
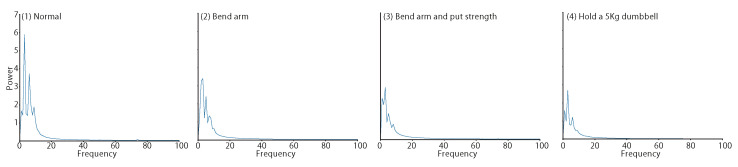
Average FFT power spectrum in four states.

Figure 5 shows the average FFT power spectrum of the 2160 samples collected for each state. The figure shows that the power spectrum was largest in the normal state (1), with a maximum value of approximately 5.9. The maximum value of the power spectrum was approximately 3.4 when the arm was bent (2), 2.9 when the arm was strained (3), and 2.7 with the arm holding a dumbbell (4). These results are the same as the order of pressure on the upper arm described above and are consistent with the intuitive magnitude of muscle activity. The amplitude of the pulse wave is smaller as the upper arm muscles contract.

The results of the state recognition of the upper arm using a random forest are as follows. The f-value of states (1), (2), (3), and (4) were 0.91, 0.82, 0.64, and 0.79, respectively. F-value, also known as F-measure, is a measure of a test’s accuracy. It is calculated from the precision and recall of the test data. The precision and recall are obtained from the following equations: TP, FN, and FP are the number of true positive, false negative, and true negative results, respectively. The F-value is the harmonic mean of precision and recall. The highest value of an F-value is 1.0, indicating perfect recognition, and the lowest value is 0 if all test data is recognized as the incorrect classes.

(5) $$\begin{array}{c} \mathrm{Precision}=\frac{\mathrm{TP}}{\mathrm{TP}+\mathrm{FN}} \end{array}$$

(6) $$\begin{array}{c} \mathrm{Recall}=\frac{\mathrm{TP}}{\mathrm{TP}+\mathrm{FP}} \end{array}$$

(7) $$\begin{array}{c} F-\mathrm{value}=\frac{2\times \mathrm{Precision}\times \mathrm{Recall}}{\mathrm{Precision}+\mathrm{Recall}} \end{array}$$

States (3) and (4) were often misrecognized. The pulse wave waveform and power spectrum of states (3) and (4) are similar. Therefore, recognition accuracy was investigated after merging states (3) and (4) as the same label (3’). The results of state recognition (1), (2), and (3’) are 0.91, 0.82, and 0.92, respectively. The accuracy of state (3’) improved by 10+%. From these results, it can be inferred that the amount of muscle activity in (4) was equivalent to (3), even if the arm was only strengthened.

### 4.2. Evaluation for Muscle Activity Recognition

This section evaluates the performance of the proposed methods for muscle activity recognition.

#### 4.2.1. Setup

Five subjects (all males, 22–25 years) joined the experiment. The subjects attached a PPG sensor to the right wrist while compressing the upper arm with a tourniquet. Pulse wave data were collected during four states same as in the preliminary experiment. Each state was maintained for 10 s, after which the arm was returned to state (1), and then maintained again for 10 s. The procedure was repeated 30 times for 10 s in each state, and a total of 100 min of data (10 s × 30 times × 4 states × 5 persons = 100 min) was collected. The power spectrum for the collected data was calculated over a 2-s sliding window with 50% overlap. A total of 1080 samples were prepared per subject. The same label (3’) was used for states (3) and (4) and the three classes were recognized in 10-fold-cross-validation for each subject.

#### 4.2.2. Result

The f-value of muscle activity recognition for each subject is shown in Table 1. In this result, training and test data came from the same subject. Focusing on the differences among the three classes, the accuracy of (1) and (2) is lower than that of (3’), especially for subjects A, B, and C. This is because (1) and (2) are misrecognized by each other. There are individual differences in pulse wave changes caused by simply bending the arm. On the other hand, the F-value of state (3’) was 0.9+ for all subjects, indicating that the state in which muscles are being used can be recognized with high accuracy.

The user-independent recognition accuracy of muscle activities for each subject is shown in Table 2. In this result, training data and test data came from different subjects, i.e., the data for subject A was recognized with the model that has learned all the data for subjects B, C, D, and E. The recognition accuracy of each subject was significantly lower than that of the user-dependent results. In all subjects, many samples were recognized as (3’), resulting in low recognition accuracy. In particular, subject C had all the samples recognized as (3’). This is because the amplitudes of pulse waves (1) and (2) of subject C were smaller than those of the other subjects and similar to those of (3’) of the other subjects. From these results, it is considered that the model created by the proposed method is user-specific and difficult to apply to other users because the amplitude of the pulse wave and the muscle mass of each subject differ.

### 4.3. Evaluation for EMG Estimation

This section evaluates the performance of the proposed methods for EMG estimation.

#### 4.3.1. Setup

Five subjects same as in the previous experiment joined the experiment. The subjects attached a PPG sensor to the right wrist and an EMG sensor to the biceps brachii muscle while compressing the upper arm with a tourniquet. Pulse wave data and EMG data were collected in four states. The procedure and amount of data collection are the same as muscle activity recognition. Thirty 10-s segments were divided into 27 segments for training and 3 for testing. A CNN is trained using a pair of pulse wave data and EMG data as training data and constructed a model to regress the RMS of EMG from pulse wave data.

#### 4.3.2. Result

**Figure 6:**
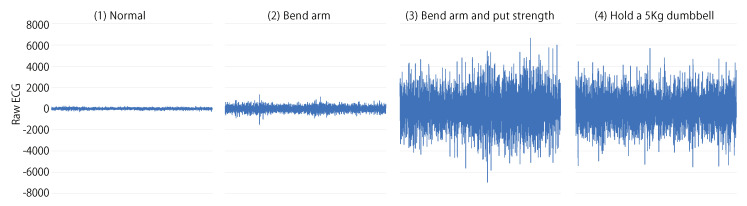
An example of raw ECG values in four states.

The average RMS of the four states for each subject is shown in Table 3. The average RMS is the average r(T) calculated for each state. We expected that the RMS would be the smallest for (1), next larger for (2), and the largest for (3) and (4). For subject E, there was no significant difference between (3) and (4), but for other subjects, the value of (4) was 1.2 to 1.8 times higher than that of (3). The RMS of subject A became smaller in (2) than in (1) because the RMS of (1) was abnormally large due to the noise. For all subjects except subject A, the RMS of (2) was larger than that of (1). Figure 6 shows the raw ECG values for one of the subjects in four states. For the figure, the amplitude of (1) is smaller than (2) and the amplitude of (2) is smaller than (3). There is no significant difference between (3) and (4).

The error rate (L1-loss divided by RMS) for each subject is shown in Table 4. Subjects A, B, C, D, and E show average error rates of 17.1%, 23.8%, 27.7%, 15.5%, and 24.8%, respectively. The difference of RMS in states (1) and (3) ranges from 839 to 1897 from Table 3 and RMS in (4) is higher than that of (3), therefore the RMS can be regressed within a precision that can discriminate whether the muscle is used or not.

## 5. Discussion

This section discusses the challenges of the currently proposed method.

### 5.1. User Dependency

The model created by the proposed method is user-dependent. Since the pulse wave changes significantly after eating or exercising, there is a possibility that the user-dependent model cannot be recognized. Changes in the user’s muscle mass may affect recognition accuracy. In addition, since the tourniquet worn on the upper arm was not removed during the experiment, the effect of the deviation of the tourniquet position was not investigated.

### 5.2. Relation of Compression and Pulse Wave

The resolution of muscle activity estimation may change depending on the pressure at which the tourniquet tightens the upper arm. For example, if the artery is tightened to the extent that it becomes congested under normal conditions, neither state recognition nor muscle activity regression can be performed because pulse wave changes do not appear during muscle activity. Conversely, if the pressure to tighten the upper arm is too small, the pulse wave will not change during muscle activity.

### 5.3. Noise of PPG Measurement

In the evaluation experiment, the subjects held a 5 kg dumbbell, which was a light burden on the male subjects. The vibration of the arm and hand increases and pulse wave measurement will be difficult as the dumbbell gets heavier. In addition, pulse waves were measured while the subjects were stationary for 10 s, but if the arm was moved quickly, the pulse wave sensor would shift, and pulse wave measurement would become difficult. Therefore, it is necessary to compensate for vibrations in the pulse wave data caused by arm movement and force. A method for estimating and correcting sensor misalignment in wearable sensing has been proposed so far [32], and it may be possible to measure pulse waves even when arm movement is large by applying these methods.

### 5.4. Load on the Body

Compression of blood vessels causes temporary congestion from the upper arm to the end of the hand. We did not consider the load on the body when the blood vessels were compressed. During the experiment, breaks were taken periodically so the subject would not be overloaded. Further investigation is needed when the compression is continued for a long period of time.

## 6. Conclusions

This paper assumes that the pulse wave (PPG: Photoplethysmography) sensor is worn on the user’s wrist to measure the heart rate. The user also attaches an elastic band to the upper arm, and when the user exerts a force on the arm, the muscles of the upper arm contract. The arteries are then constricted, and the pulse wave measured at the wrist becomes weak. By focusing on the phenomena, we proposed a method that recognizes the muscle activity states of the arm and a method that estimates EGM using pulse wave data obtained smartwatch PPG sensor. From the evaluation experiment with five subjects, three types of muscle activity were recognized with 80+%, and sEMG was estimated with approximately 20% error rate. The proposed method can be used to count muscle training. A more accurate logging system than the accelerometer approach is achieved by using only the pulse wave since the acceleration value fluctuates depending on arm motion unrelated to training.

## Figures and Tables

**Table 1:** F-value of muscle activity recognition.

Arm State	Subject					
	A	B	C	D	E	Average
(1) Normal	0.72	0.72	0.79	0.78	0.87	0.776
(2) Bend arm	0.63	0.65	0.68	0.81	0.76	0.706
(3’) Put strength & hold dumbbell	0.90	0.94	0.93	0.95	0.90	0.924
Average	0.75	0.77	0.80	0.85	0.84	

**Table 2:** Results of muscle activity recognition (user-independent).

Arm State	Subject					
	A	B	C	D	E	Average
(1) Normal	0.49	0.31	0.00	0.07	0.18	0.21
(2) Bend arm	0.27	0.34	0.00	0.15	0.04	0.16
(3’) Put strength & hold dumbbell	0.78	0.72	0.67	0.69	0.69	0.71
Average	0.51	0.46	0.22	0.30	0.30	

**Table 3:** RMS of EMG for four muscle activity states.

Arm State	Subject				
	A	B	C	D	E
(1) Normal	961	422	159	342	59
(2) Bend arm	443	848	247	348	223
(3) Put strength	2246	1261	1129	2239	1394
(4) Hold dumbbell	2716	2351	1924	3506	1422

**Table 4:** Error rate [%] of RMS for four muscle activity states.

Arm State	Subject				
	A	B	C	D	E
(1) Normal	20.1%	22.2%	22.5%	13.6%	32.2%
(2) Bend arm	18.7%	20.2%	27.0%	14.1%	37.0%
(3) Put strength	11.1%	14.3%	27.4%	15.7%	17.1%
(4) Hold dumbbell	17.8%	67.5%	46.7%	20.0%	16.4%
Average	17.1%	23.8%	27.7%	15.5%	24.8%

## Data Availability

The data are not publicly available due to using biometric information such as ECG signal and pulse wave.
